# Integrative analysis of physiological responses to high fat feeding with diffusion tensor images and neurochemical profiles of the mouse brain

**DOI:** 10.1038/s41366-021-00775-9

**Published:** 2021-02-11

**Authors:** Irene Guadilla, Blanca Lizarbe, Laura Barrios, Sebastián Cerdán, Pilar López-Larrubia

**Affiliations:** 1 Instituto de Investigaciones Biomédicas “Alberto Sols” CSIC-UAM, Madrid, Spain; 2 Centro Técnico de Informática CSIC, Madrid, Spain

**Keywords:** Obesity, Obesity

## Abstract

**Background:**

Obesity proceeds with important physiological and microstructural alterations in the brain, but the precise relationships between the diet and feeding status, its physiological responses, and the observed neuroimaging repercussions, remain elusive. Here, we implemented a mouse model of high fat diet (HFD) feeding to explore specific associations between diet, feeding status, phenotypic and endocrine repercussions, and the resulting microstructural and metabolic alterations in the brain, as detected by diffusion tensor imaging (DTI) and neurochemical metabolic profiling.

**Methods:**

Brain DTI images were acquired from adult male C57BL6/J mice after 6 weeks of HFD, or standard diet (SD) administrations, both under the fed, and overnight fasted conditions. Metabolomic profiles of the cortex (Ctx), hippocampus (Hipc), and hypothalamus (Hyp) were determined by ^1^H high-resolution magic angle spinning (HRMAS) spectroscopy, in cerebral biopsies dissected after microwave fixation. Mean diffusivity (MD), fractional anisotropy (FA) maps, and HRMAS profiles were complemented with determinations of phenotypic alterations and plasma levels of appetite-related hormones, measured by indirect calorimetry and multiplex assays, respectively. We used *Z*-score and alternating least squares scaling (ALSCAL) analysis to investigate specific associations between diet and feeding status, physiological, and imaging parameters.

**Results:**

HFD induced significant increases in body weight and the plasma levels of glucose and fatty acids in the fed and fasted conditions, as well as higher cerebral MD (Ctx, Hipc, Hyp), FA (Hipc), and mobile saturated fatty acids resonances (Ctx, Hipc, Hyp). *Z*-score and ASLCAL analysis identified the precise associations between physiological and imaging variables.

**Conclusions:**

The present study reveals that diet and feeding conditions elicit prominent effects on specific imaging and spectroscopic parameters of the mouse brain that can be associated to the alterations in phenotypic and endocrine variables. Together, present results disclose a neuro-inflammatory response to HFD, characterized primarily by vasogenic edema and compensatory responses in osmolyte concentrations.

## Introduction

Obesity and overweight are thought to develop from unhealthy life style habits, combining the exacerbated consumption of diets rich in fats and sugars with sedentary behaviors [1]. These circumstances lead, ultimately, to impairments in the regulation of the cerebral mechanisms controlling global energy balance and addictive behavior [2], favoring a phletora of life-threatening comorbidities which have reached pandemic proportions worldwide [3, 4].

Initial evidences suggested that obesity developed from endocrine alterations in perypheral signals, essentially insulin and leptin, controlling the balance between hypothalamic orexigenic and anorexigenic responses, mainly through the melanocortin pathway [5, 6]. Briefly, long-term increases in the plasmatic levels of leptin or insulin, resulted in insulin or leptin resistance, paving the way to diabetes or fat accumulation [7]. More recently, dietary saturated fatty acids (SFA) have been shown to permeate through the blood–brain barrier (BBB) triggering an NFκB-orchestrated neuroinflamatory response in the hypothalamus, and other appetite regulating regions in the brain, that precedes weight gain [8–10]. Moreover, considerable evidence accumulated, revealing important morphological and metabolic alterations in the brain of obese individuals, as well as in animal models of obesity. These included in general, gray matter reductions [11] and cognitive impairment [12], as well as increases in tricaboxylic acid (TCA) cycle flux, anaplerosis, and GABA production [13, 14].

Magnetic resonance imaging (MRI) methods have contributed considerable progress to the understanding of global energy metabolism in the brain, and its disturbances in vivo [15]. Briefly, diffusion weighted imaging (DWI) revealed alterations of brain microstructure in rodents and humans subjected to feeding/fasting paradigms [16–19]. In addition, ^1^H high-resolution magic angle spinning (HRMAS) and in vivo ^13^C magnetic resonance spectroscopy, unveiled considerable changes in the cerebral neurochemical profile in mice subjected to high fat diet (HFD), or increases in hypothalamic TCA cycle activity and GABAergic neurotransmission, in leptin-deficient Ob/Ob mice [14]. Diffusion tensor imaging (DTI) approaches have added, recently, considerable potential to evaluate white matter microstructural changes in obese humans, but their application to animal models of obesity, more specifically to mouse models, remained to be explored [20].

Animal models have played a fundamental role in the understanding of the physio-pathological mechanisms underlying obesity and the development of therapeutic interventions [21]. In particular, a large variety of genetic models, including leptin-deficient Ob/Ob mice, or obese Zucker rats, have been crucial to reveal some of the molecular and cellular signatures involved in obesity. However, obesity and overweight result most frequently, from pleiotropic polygenic interactions and environmental determinants such as the diet type, or the level of physical activity [1, 22]. Diet-induced obesity models appear, from this perspective, more adequate to evaluate dynamically the effects of excessive caloric intake during obesity development [1, 23]. Among these, the HFD model has been primarily used in rodents [24], presenting higher body weight gain than controls [25], increased glucose and insulin levels [26], and insulin and leptin resistance [27]. Moreover, HFD models allow to investigate feeding/fasting paradigms superimposed to the dietary manipulations, an acute nutritional intervention currently considered to promote activation of a critical neuro-inflammatory profile, that precedes weight gain [28]. However, despite the wealth of information accumulated in HFD rodent models, no studies to our knowledge, have addressed from an integral perspective, the plethora of specific associations occurring among diet and feeding conditions, its phenotypic or endocrine consequences, and the resulting alterations in cerebral imaging and metabolic parameters.

On these grounds, we aimed here to contribute an integrative analysis of the relationships between physio-pathological variables and the corresponding imaging and spectroscopic parameters in the brain of C57BL6/J mice subjected to HFD.

## Methods

### Animals and experimental design

Eight-week old C57BL6/J healthy mice (*n* = 60, 22 ± 2 g) were randomly divided in two groups (*n* = 30 each) and fed for 6 weeks, either with a standard diet (SD) (A04, SAFE Augy, France, 2900 kcal/kg), or a high-saturated-fat (60%) diet HFD (260HF, SAFE Augy, France, 5505 kcal/kg). After 6 weeks of diet diversification, mice (*n* = 20 HFD, *n* = 20 SD) fed ad libitum, or 16 h fasting, were subjected to noninvasive MRI acquisitions (*n* = 10 HFD, *n* = 10 SD), or phenotyping evaluation (*n* = 10 HFD, *n* = 10 SD) in an automatically monitored cage system (Phenomaster, TSE Systems GmbH, Germany) (Supplementary Fig. 1).

Animals were euthanized with a high-power microwave fixation system focused in the brain (TMW-4012C 5 kW, Muromachi Kikai Co. Ltd., Japan). Fixed brains were then isolated from the skull, the hypothalamus (Hyp), hippocampus (Hipc), and temporal cortex (Ctx) dissected, and the samples kept at −80 °C for subsequent HRMAS acquisitions. Design and reporting adhered to the ARRIVE guidelines [29].

### Physiological characterization

#### Phenotyping

Animals were individually evaluated in a phenotyping system that monitored automatically food and water consumption, as well as spontaneous respiratory and motor activities [30].

#### Endocrine profile

Leptin, insulin, ghrelin, PYY, glucagon, and GLP-1 concentrations were determined in plasma samples obtained from HFD or SD mice, under the fed or fasted conditions, using the MILLIPLEX MAP Mouse Metabolic Hormone Magnetic Bead Panel (MMHMAG-44K Multiplex-assay), using the Luminex^®^ platform (see Supplementary information).

### Magnetic resonance imaging and spectroscopy

#### Diffusion tensor imaging

Anesthetized animals underwent MRI acquisitions on a Bruker 7T BioSpec system (Bruker Biospin, Ettlingen, DE), after determining blood glucose levels with a standard glucometer (Accu-Chek^®^Aviva, Roche) in samples extracted from the tail vein. MRI studies acquired T_2_ morphological images to identify the section containing hypothalamus, and a set of diffusion weighted images were acquired using a DTI sequence [31], with diffusion gradients applied in six directions. Diffusion images were analyzed with a homemade software package by fitting, voxel by voxel, the diffusion signal decay to a mono-exponential model, yielding mean diffusivity (MD) and fractional anisotropy (FA) maps [32]. Regions of interest including Ctx, Hipc, and Hyp were manually selected from the parametric maps by using ImageJ software (National Institutes of Health, Bethesda, MD, USA, ImageJ) overlaid over a mouse brain atlas [16, 19].

#### ^1^H high-resolution magic angle spinning spectroscopy

Small biopsies of Hyp, Hipc, and Ctx were used to obtain ^1^H HRMAS spectra using a Bruker AVANCE 11.7T equipped with a HRMAS accessory. Studies were processed and quantified with LCModel (linear combination of model spectra) [33], which fits the HRMAS spectrum as a linear combination of individual model spectra contained in a database of cerebral metabolites (see additional experimental details in Supplementary information).

### Statistics

In all cases, data that did not achieve the quality requirements according to SNR in MR images and restriction coefficients in fitting processing, as well as outliers, were excluded from the statistical analyses.

#### Univariate analysis

Physiologic and phenotyping statistics were calculated using GraphPad Prism 6 (GraphPad Software, San Diego, CA). Body weight and blood glucose differences were evaluated, where indicated, with multiple unpaired *t*-tests or two-tailed paired *t*-tests.

#### Generalized linear model

Statistical analyses of MRI data, HRMAS metabolites, and blood biochemical parameters were performed using the IBM SPSS package (IBM SPSS Statistics for Windows, Version 25.0. Armonk, NY, IBM Corp). Evaluations of MRI-derived parameters were performed using the generalized linear model and generalized estimating equations (GEE), which allows for the analysis of repeated measures [34]. Data from HRMAS and blood analyses were evaluated with a linear mixed model. In all cases, *p* values < 0.05 were considered statistically significant.

#### *Z*-score evaluation

*Z*-scores were calculated from the mean values of each variable (MRI parameters, relative concentration of brain metabolites from HRMAS, plasmatic hormone levels, and phenotypic data) from the four experimental conditions (SD-fed, SD-fasted, HFD-fed, HFD-fasted), respectively, regarding their respective sample mean between the four conditions. Four independent bar graphs, corresponding to four experimental conditions, contain the *Z*-scores from all individual variables, allowing to overview of the patterns of change among them.

#### Alternating least squares scaling (ALSCAL)

ALSCAL is a nonmetric multidimensional scaling method that finds the structure of a set of distance measures between objects or cases, by assigning observations to specific locations in a new conceptual space (usually two-dimensional), such that the distances between points in the new space match the given similarities/dissimilarities as closely as possible. In this study, it was firstly calculated the Euclidean distance between the *Z*-scores of the original variables (MD, FA, GABA, Glu, Gln, Glc, Myo, NAA, Cr, GPC + Cho, Lip09, Lip13, relation Lip13/09, glucagon, insulin, leptin, PYY, weight, glucose, drink, food, RER, DistK, and calories), as measured in the four experimental conditions (SD-fed, SD-fasted, HFD-fed, HFD-fasted). We then transformed the multidimensional Euclidean distance space into a new two-dimensional space, using a Euclidean model, yielding a conceptual representation of the relationships among all variables and the corresponding diet/feeding conditions (additional information in Supplementary information). In this representation, two variables with similar behavior are depicted in the same area, while variables with distinct trends are separated by longer distances.

## Results

### Physiological and imaging characterization of the animal model

**Fig. 1:**
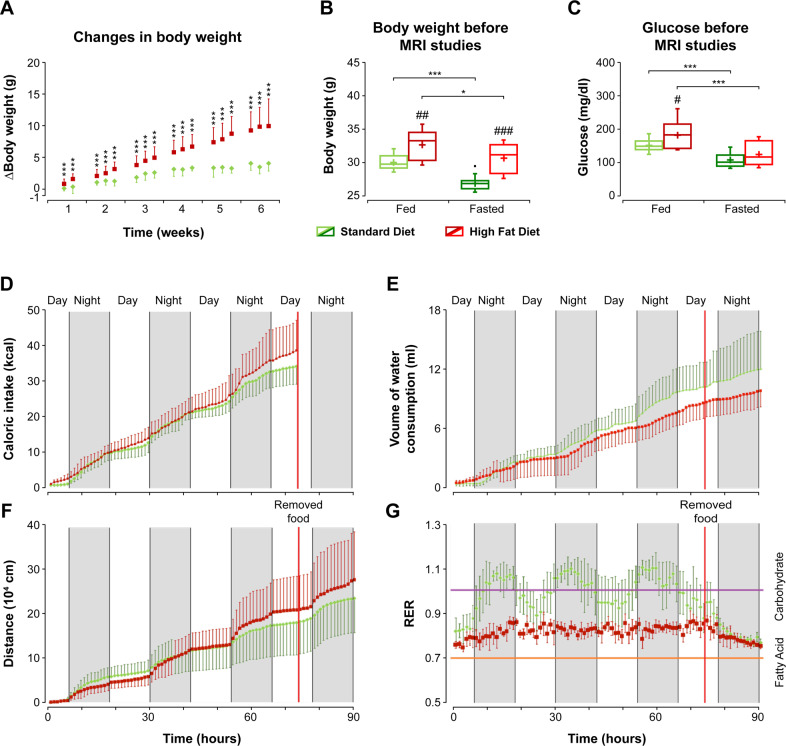
Physiological characterization of the C57BL6/J mouse model receiving high fat, or standard diets. **A** Time course of changes in body weight along HFD (red), or SD (green) administrations; **B** box plots of body weights before imaging sessions under fed or fasted conditions; **C** box plots of blood glucose levels before the imaging sessions under fed or fasted conditions. In each box plot, the central bar indicates the median, and the bottom and top edges refer to the 25th and 75th percentiles, respectively. The upper and lower limits of the box extend to the most extreme data points not considered outliers, which are plotted individually using the “•” symbol. Time course (90 h) of phenotyping variables from mice subjected to HFD or SD diets in the Phenomaster^®^ system (**D** caloric intake; **E** water consumption; **F** locomotor activity (measured as distance walked), **G** RER values). *^/#^*p* < 0.05, ^##^*p* < 0.01, ***^/###^*p* < 0.001.

Animals fed with HFD depicted larger increases in body weight than mice receiving SD (Fig. 1A). Notably, these differences became significant after the first week of diet diversification, reaching 10–14% higher values after the 6-week period. Body weight values measured before MRI performance (Supplementary information, Table 1) were also significantly higher in HFD mice as compared to SD animals, both in the fed and fasting conditions (Fig. 1B). In both cohorts, overnight fasting caused a statistically significant decrease in body weight, more pronounced in the SD group (11%), than in HFD animals (6%). Blood glucose levels measured prior to the MRI acquisitions were significantly higher in the fed state of HFD animals (*p* < 0.05), and decreased upon fasting (*p* < 0.05) (Fig. 1C) (Supplementary information, Table 1).

Mice evaluations in the phenotyping system did not reveal significant differences between either the diets, or the feeding conditions (Fig. 1D, F). However, the respiratory exchange ratio (RER) (VCO_2_/VO_2_) of SD mice exhibited periodical oscillations around 1, with RER values being higher at night (active period) and lower during the day (resting period) (Fig. 1G). Notably, food removal eliminated the above-mentioned RER fluctuations, decreasing RER values until almost 0.7. The circadian oscillations were not observed in the animals fed with HFD for 6 weeks, with RER values approaching 0.7, during the complete experimental period.

**Fig. 2:**
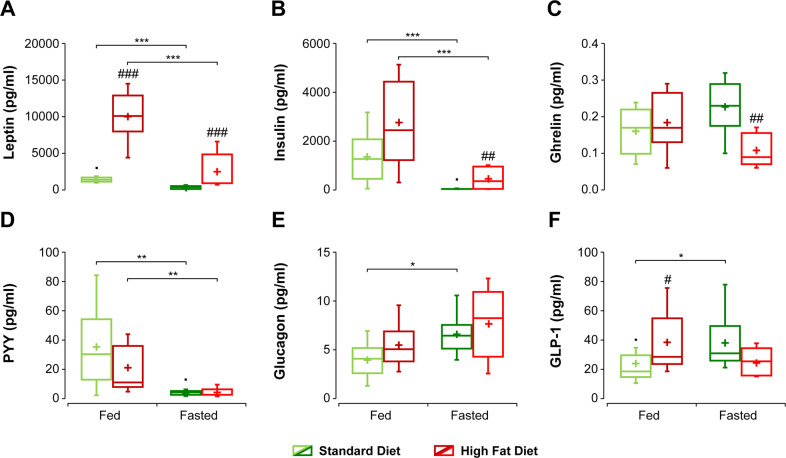
Endocrine profiles in the plasma of C57BL6/J mice receiving high fat or standard diets, under fed or fasted conditions. Appetite-related hormones were determined (pg/ml) with multiplex assays in plasma samples, obtained and processed as indicated in “Methods” (and Supplementary information). **A** leptin, **B** insulin, **C** ghrelin, **D** PYY_3-36_, **E** glucagon, **F** glucagon-like peptide 1 (GLP1). Box plots are represented as indicated in the legend to Fig. 1. *^/#^*p* < 0.05, **^/##^*p* < 0.01, ***^/###^*p* < 0.001.

Hormonal profiles determined in blood samples (Fig. 2A–F) revealed that leptin levels were significantly higher in the HFD than SD groups both in the fed and fasted conditions (Fig. 2A). The fed state showed significantly increased insulin concentrations in both diet groups (Fig. 2B). Ghrelin concentration showed a tendency to increase upon fasting in the SD group, but the opposite was observed in the HFD group, with SD-fasted values significantly lower than HFD-fasted ones (Fig. 2C). PYY levels decreased significantly upon fasting in both diet cohorts (Fig. 2D). On the contrary, glucagon increased upon fasting in both diet groups, with the increases in SD animals reaching significance (Fig. 2E). Finally, GLP-1 levels augmented significantly after overnight fasting in SD, but not in HFD animals (Fig. 2F).

**Fig. 3:**
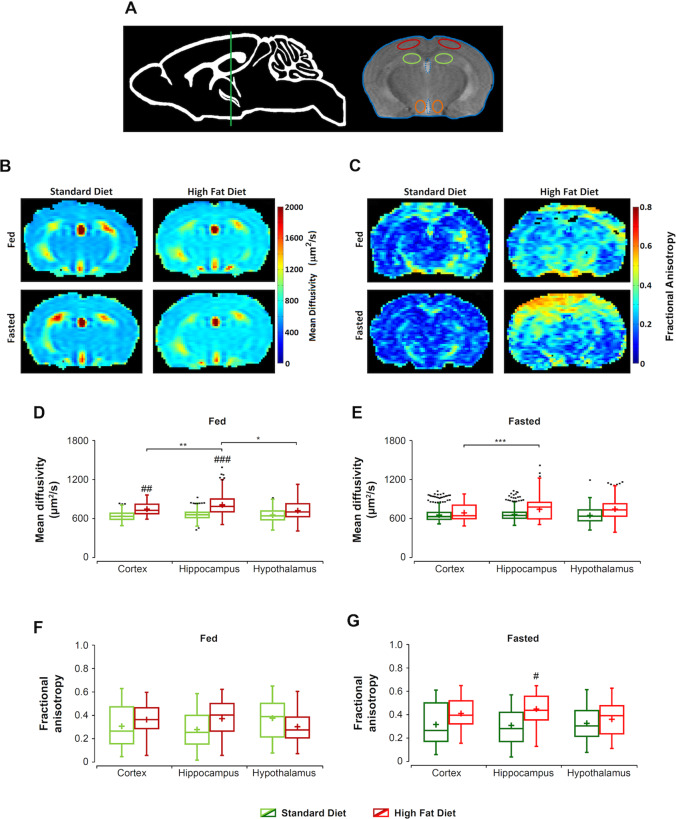
Mean diffusivity and fractional anisotropy maps through the investigated brain section of C57BL6/J mice receiving high fat or standard diets, under the fed or fasted conditions. **A** Anatomical location of the slice selected, including perimeters of investigated ROIs. Temporal cortex (red), hippocampus (green), and hypothalamus (orange). **B** Representative parametric maps of mean diffusivity (MD) from mice fed HFD or SD diets, under the fed or fasted conditions, respectively. The side color bar indicates the range of values from low (blue) to high (red). **C** Representative parametric maps of fractional anisotropy (FA) from mice fed HFD or SD diets, under the fed or fasted conditions, respectively. The range of values is represented in the side color bar, from lower (blue) to higher (red) values. **D** Box plots of cerebral MD values from the cortex, hippocampus, and hypothalamus of mice receiving HFD (red), or SD (green) diets, under the fed condition. **E** Box plots of cerebral MD values from the cortex, hippocampus, and hypothalamus of mice receiving HFD (red), or SD (green) diets, under the fasted condition. **F** Box plots of cerebral FA values from the cortex, hippocampus, and hypothalamus of mice receiving HFD (red), or SD (green) diets, under the fed condition. **G** Box plots of cerebral FA values from the cortex, hippocampus, and hypothalamus of mice receiving HFD (red), or SD (green) diets, under the fasted condition. Box plots are represented as in Fig. 1. *^/#^*p* < 0.05, **p < 0.01, ***^/###^*p* < 0.001.

Figure 3 summarizes the effects of HFD or SD, and the corresponding fed or fasted conditions, on parametric maps of MD of the mouse brain. Figure 3A illustrates the anatomical localization of the slice selected and different cerebral structures investigated (Supplementary information). Although parametric maps show a shift to light blue colors in HFD animals, consistent with increased MD (Fig. 3B), comparisons of average MD values through the whole brain in the slice did not reach statistical significance (Supplementary information, Table 2). However, statistically significant differences were detected when different regions were considered. Particularly, the GEE statistical analysis revealed significant effects of diet (*p* = 0.002), region (*p* < 0.001), and the product diet × region × condition (*p* < 0.001) on the MD coefficients. Pairwise comparisons showed statistically remarkable differences between MD values of the two diet groups in the fed state, in the Ctx and Hipc regions. The representation of MD values in box plots (Fig. 3D) confirms the higher MD values in HFD animals, as compared to SD mice. Besides, MD of HFD-fed animals was significantly higher in Hipc than in Ctx and Hyp. However, pairwise comparisons between SD-fasted and HFD-fasted did not reveal significant differences (Fig. 3E), except a significant increase in HFD-fasted Hipc, as compared to HFD-fasted Ctx.

Figure 3 also shows results from the assessment of FA, with representative cerebral maps from mice subjected to SD or HFD, under both feeding conditions (Fig. 3C). Comparisons of average FA values through the whole brain did not show significant differences (Supplementary Information, Table 2). However, the statistical assessment of the FA coefficients revealed a significant effect of the product diet × region × condition (*p* = 0.005), while diet, region, and feeding condition individually did not induce significant FA changes. The corresponding box plots (Fig. 3F, G) revealed in general a tendency to increase FA in HFD animals, which became significant in the Hipc of fasted animals only.

**Fig. 4:**
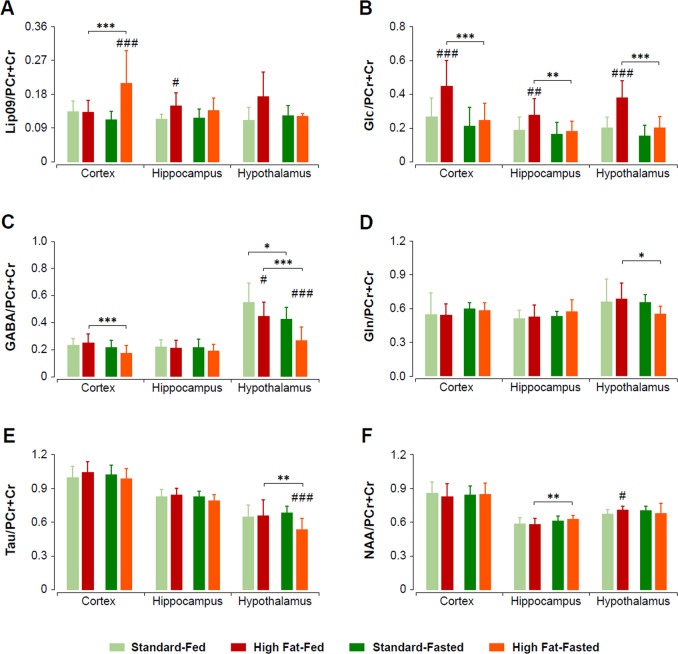
HRMAS neurochemical profiles of biopsies of the cortex, hippocampus, and hypothalamus from C57BL6/J mice receiving high fat or standard diets, under the fed or fasted conditions. **A** Lipid methyl (0.9 ppm), **B** glucose (Glc), **C** GABA, **D** glutamine (Gln), **E** taurine (Tau), **F** NAA. All values are normalized to the total creatine (PCr + Cr) content. Diet and feeding conditions are represented using the color code of the previous figures. *p < 0.05, **p < 0.01, ***p < 0.001.

Figure 4 summarizes the neurochemical profiles from brain biopsies of hypothalamus, hippocampus, and cortex, under the different diets and feeding conditions investigated, as obtained by ^1^H HRMAS spectroscopy (Supplementary Fig. 2). Statistical analysis revealed significant changes within diets, feeding status and anatomical regions, involving mainly GABA (Ctx and Hyp), Gln (Hyp), GPC + Cho (Hyp), NAA (Hipc and Hyp), Tau (Hyp), Glc (Ctx, Hipc, and Hyp), Glu (Ctx and Hyp), and Lip09 (Ctx and Hipc) concentrations (Fig. 4).

More specifically, HFD feeding increased the relative concentrations of mobile lipid methyl (at 0.9 ppm, Lip09) groups in all cases, with more pronounced effects in the Ctx and Hyp under fasting conditions (Fig. 4A). The ratio between lipid resonances (Lip13/Lip09) remained constant with diet and feeding condition (not shown), revealing no significant change in the average length of the fatty acyl chains. Moreover, no resonances from the vinyl protons were detected in the 5–6 ppm region (Supplementary Fig. 2), revealing that the Lip09 and Lip13 resonances correspond to SFA. Glucose (Glc) levels were significantly increased in HFD feeding mice as compared to standard diet, and decreased in all regions and both cohorts (Fig. 4B). Relative GABA concentration decreased significantly during fasting in the Ctx and Hyp of the HFD cohort, and in the Hyp of SD animals. In this region, also significant inter diet group differences were detected in both feeding conditions (Fig. 4C). Glutamine (Gln) (Fig. 4D) and glutamate (not shown) concentration decreased significantly during fasting in the HFD mice Hyp. Similarly, GPC + Cho experienced a significant decrease only in the hypothalamus of HFD animals (not shown). Taurine (Tau) concentration, which was similar among diet groups in the fed state, decreased significantly with fasting in the HFD hypothalamus, remaining remarkably lower than the corresponding fasted SD counterparts (Fig. 4E). Interestingly, NAA hypothalamic concentration was significantly different in the sated state, with higher concentrations being detected on HFD animals and NAA values increasing with fasting in the Hipc of HFD mice (Fig. 4F).

### Statistical analysis

**Fig. 5:**
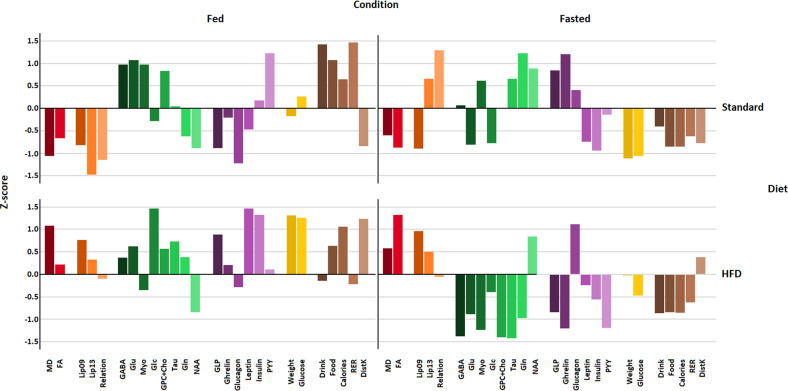
*Z*-score plots of observed variables from the brain of mice subjected to SD or HFD diets, under fed/fasted conditions. MD (mean diffusivity), FA (fractional anisotropy), Lip09 (methyl resonances from saturated fatty acids), Lip13 (methylene resonances from saturated fatty acids), Relation (Lipid09/13 ratio), GABA (γ‐aminobutyrate), Glu (glutamate), Myo (myo-inositol), Glc (glucose), GPC + Cho (glycerophosphocholine + choline), Tau (taurine), Gln (glutamine), NAA (N-acetyl-aspartic acid), GLP (plasma glucagon-like peptide 1), Ghrelin (plasma ghrelin), Glucagon (plasma glucagon), Leptin (plasma leptin), Insulin (plasma insulin), PYY (plasma PYY_3-36_ peptide), Weight (body weight), Glucose (plasma glucose), Drink (water consumed), Food (food consumed), Calories (caloric intake), RER (respiratory exchange ratio), DistK (motor activity as distance walked).

The *Z*-scores method normalized the investigated variables in terms of their standard deviations, identifying those that present similar, or opposite, responses to HFD or SD, or to feeding/fasting conditions (Fig. 5). *Z*-scores for MD, FA, and Lip09 were negative for SD mice but positive for HFD animals, with close to (−1), or to (+1) values, respectively, in an independent manner from the feeding condition. Thus, these imaging and spectroscopic variables provide adequate parameters to distinguish the cerebral effects of both diets, independently of the feeding status. On the contrary, Myo showed positive *Z*-scores for SD mice but negative for HFD animals, both under feeding or fasting conditions, offering additional criteria to identify the different diets administered, from the observed neurochemical profiles ex vivo.

Interestingly, other variables changed the sign of corresponding *Z*-scores depending only on the feeding/fasting conditions, independently of the diet consumed. Such is the case for the plasma levels of glucose and insulin, which depicted high-positive *Z*-scores on the fed state, and negative values after 16 h fasting. This provides a measure of confidence on the performance of this methodology, confirming the dominant role of these variables in the discrimination between the feeding conditions.

Additionally, *Z*-scores for glutamate, PYY, food, and calories were negative in the fasted measurements, but positive in the fed, or NAA and glucagon with positive values at fasting but negative at feeding. Gln or ghrelin (to a lesser extent) depicted a crossed-response trend of *Z*-scores, being negative for the SD-fed and HFD-fasted states, and positive in the HFD-fed and SD-fasted conditions. This reveals that the *Z*-score analysis is able to detect additional biomarkers defining the feeding or fasting conditions.

**Fig. 6:**
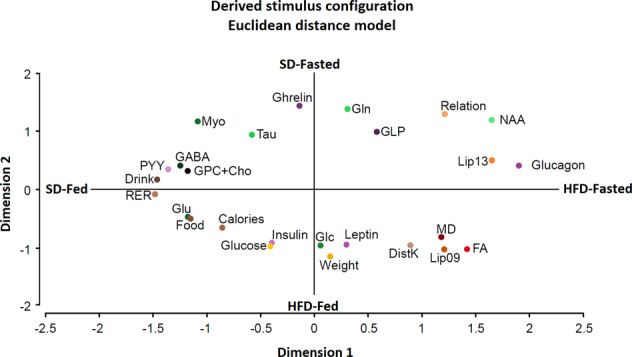
Alternating least squares scaling (ALSCAL) diagram relating phenotyping and endocrine variables with imaging parameters and neurochemical profiles, following HFD or SD administrations, under the fed or fasted conditions. Variables are displayed in the new bi-dimensional space, with coordinates defined through the transformation of the Euclidean distance between variables. Abbreviations and colors are those of Fig. 5.

ALSCAL analysis led to a new two-dimensional space representation integrating all investigated variables and their specific associations to the four possible combinations of diet and feeding conditions (Fig. 6). In this space, each variable is represented with respect to the others by the transformation of the Euclidean distance between each pair of variables. This makes possible to group those variables that are at close range, or represent better, the four diet/feeding conditions. In particular, glucagon, Lip13, and NAA defined a cluster that is more closely associated to the HFD-fasted situation, while glucose, insulin, and weight clustered closer to HFD-fed. Similarly, GABA, PYY, drink, RER, and GPC + Cho were more closely associated to SD-fed, while ghrelin and Gln lied to SD-fasted. Similar ALSCAL analyses were performed in Hyp, Hipc, and Ctx regions showing analogous general trends, but also some specific characteristics (Supplementary Fig. 3). In particular, Glc, insulin, and glucose or weight and leptin small distance was maintained in all three structures. RER, drink, and PYY also presented adjacent positions in cortex, hippocampus, and hypothalamus. In the cortical region, food, Glu, and calories and the lipid ratio (Relation), Myo, Lip13, and Gln depicted close positions. In the hippocampus, MD, Lip09, and DistK; leptin, and weight; glutamate and calories; RER and ghrelin groupings exhibited small distances between their variables. Finally, hypothalamus presented close positions in the groups formed by Lip09, Lip13, and lipid relation; GLP and NAA; Tau and Gln; GPC + Cho and Glu; Myo and GABA.

## Discussion

### Physiological and neuroimaging characterization of the HFD mouse model

HFD feeding increased significantly caloric intake and body weight, together with hyperglycemia, hyperinsulinemia, and hyperleptinemia under the fed condition, which decreased appreciably upon fasting. Together, these results revealed that the model used, reproduced the expected behavioral and endocrine adaptations [35–42], as well as the development of an insulin and leptin resistant phenotype, also observed in obese individuals [42]. Similarly, the feeding/fasting paradigm resulted in phenotypic and endocrine responses similar to those described in earlier studies [43], providing a solid frame supporting further analyses.

DTI provides information on the alterations of the mouse brain microstructure as revealed by the MD and FA parameters. Cerebral MD measurements represent the net balance of Brownian water movements through the extracellular and intracellular compartments of the brain [44]. In this sense, net increases in MD reveal a dominant contribution of fast diffusion movements through the extracellular space, normally associated to alterations of BBB integrity and vasogenic edema. On the contrary, net decreases in MD reflect predominance of slower intracellular diffusions, typically associated with neurocellular swelling episodes, or cytotoxic edema. Our results revealed that HFD induced net increases of MD in the Ctx and Hipc of fed C57BL6/J mice, a difference that was not maintained in the fasting state. Since fasting has been shown to decrease apparent diffusion coefficient (ADC) values in these regions [16, 19], the reversal of increased MD in the cortex of HFD mice under fasting, reveals the effective cancellation of increased MD values derived from HFD, with the decreases in MD under the fasted condition. This compensatory response appears to be heterogeneous through the brain [19], providing only partial cancelation in the Hipc, where mild net increases in MD persist in the HFD-fasted condition. Notably, the absence of significant MD changes in the Hyp does not imply the lack of HFD effects in this structure but rather, an effective compensation of the opposite ADC changes induced by both diets/feeding conditions. Together, these results are consistent with previous works reporting increased diffusion coefficients and vasogenic edema during obesity, even under normal feeding regimes [20, 45, 46], while reveal for the first time to our knowledge, partial or total reversal of such increases after a fasting period.

FA measurements provide information on the anisotropic orientation of neuronal tracts through the white and gray matters [46, 47]. Present results reveal that HFD mildly increases the average anisotropic orientation of axons observed in the Ctx, Hipc, and Hyp of SD animals. In this sense, FA increases detected in all structures suggest that the corresponding neurocellular responses to HFD, result in small, albeit potentially important, changes in neuronal tract orientation in all structures investigated, reaching statistical significance only in the hippocampus of fasted animals. Our results are consistent with previous works reporting increased cerebral diffusion during obesity in humans [45] but reveal, for the first time, the response of the mouse brain to HFD under the fed or fasted conditions, as disclosed by MD and FA measurements.

The absolute quantification of HRMAS spectra provide the most reliable information of changes in metabolite concentrations. When this is not achievable, it is crucial to deal with the potential effects of the experimental conditions on the normalization process and the metabolite used to that. In our case, we opted for a relative quantification once previous studies stated the nonexistence of alterations in the total creatine content either due to HFD feeding [18] or fasting state [48]. The neurochemical profiles detected by HRMAS closely reflected the effects of different diets and feeding conditions in the mouse brain. Notably, HFD resulted in increased SFA in all regions investigated, more pronounced under fasting. SFA are known to trigger a neuro-inflammatory response mediated by binding to Toll-like receptors and the activation of the NfκB transcription cascade in the hypothalamus [49], a crucial event underlying the complex edema responses of MD and FA discussed above [50]. Remarkably, HFD induced also similar responses to diet/feeding conditions in the osmolytes Tau [51], Gln [52], and NAA of the Hyp. These reactive changes in osmolyte concentration disclose a complex and heterogeneous neuro-inflammatory response to increased SFA in the Hyp and Hipc, with significantly smaller effects in the Ctx. Finally, HFD showed reductions in the GABA concentrations [53], more pronounced in the fasted state, disclosing an important role for GABA as a novel biomarker for the different diet/feeding conditions.

### Integrative analysis of neuroimaging and physiological variables

*Z*-scores provide a convenient statistical method to investigate complex responses to a given perturbation in large collections of multiple interacting variables. Briefly, it reduces the complexity of the universe of variables by identifying those that respond similarly to the investigated diet/feeding condition. *Z*-scores data revealed that MD, FA, and lipid resonances responded to the perturbations together, as a group, suggesting that the changes in these imaging and spectroscopic variables follow a coordinated mechanism. Concerning feeding conditions, *Z*-scores for plasma insulin increased, as expected, under feeding and decreased after 16 h fasting, providing a measure of confidence on the ability of the *Z*-score method to identify correctly the variable changes, and confirming the dominant role of insulin in the discrimination between the feeding/fasting conditions [37]. Similarly, *Z*-scores for glutamate, PYY, glucose, food consumed, and caloric intake decreased after fasting, disclosing that these variables may share, with insulin, a common response mechanism to the diet administered. On the contrary, NAA showed increased *Z*-scores in fasting state. Finally, ASLCAL analysis supported the *Z*-scores results; identifying groups of variables more closely representing the SD-fed and HFD-fasted states as the most separated profiles. In particular, glucagon, NAA, and Lip13 grouped closer the HFD-Fasted condition, while GABA, PYY, GPC + Cho, drink, and RER grouped close to the SD-Fed, suggesting that these groups variables are those that define more adequately these conditions. Similarly, glucose, weight, and insulin, or ghrelin, and Gln, provided adequate biomarkers for HFD-fed, or SD-fasted situations, respectively.

## Concluding remarks

In summary, we report that the cerebral imaging parameters MD and FA, and the neurochemical profile, including mainly SFA and osmolyte variations, provide adequate biomarkers to define the effects of HFD in the brain of adult C57BL6/J mice. In particular, these imaging and spectroscopic alterations unveil a characteristic neuro-inflammatory response of the mouse brain to HFD feeding that can be modulated by fasting. Similarly, the variations in phenotypic or endocrine variables, as food and water consumption, or insulin, leptin, and glucagon, are found to provide suitable biomarkers for the feeding/fasting transition. Taken together, the present findings reveal that diet and feeding conditions elicit important effects in physiological parameters, imaging and metabolic profiles of the mouse brain, providing the specific integrative associations.

## Supplementary information

Supplementary Information
