# Supplementary material for: Integrative analysis of physiological responses to high fat feeding with diffusion tensor images and neurochemical profiles of the mouse brain
Source: Int J Obes (Lond). 2021 Feb 11;45(6):1203–14. doi: 10.1038/s41366-021-00775-9 (PMC8159736; doi:10.1038/s41366-021-00775-9)
Supplement: Supplementary file 1 — Supplementary Information [file 41366_2021_775_MOESM1_ESM.pdf]

SUPPLEMENTARY INFORMATION

Integrative Analysis of Physiological Responses to High Fat Feeding with Diffusion Tensor Images and Neurochemical Profiles of the Mouse Brain.

Irene Guadilla[1], Blanca Lizarbe[1], Laura Barrios[2], Sebastián Cerdán[1] and Pilar López-Larrubia[1]*

[1]Instituto de Investigaciones Biomédicas “Alberto Sols” CSIC/UAM, c/ Arturo Duperier 4, Madrid 28029, Spain, [2] Centro Técnico de Informática CSIC, c/Pinar 19, Madrid 28006

Corresponding author:

Pilar López-Larrubia
Instituto de Investigaciones Biomédicas “Alberto Sols” CSIC/UAM
c/ Arturo Duperier 4
Madrid 28029
Spain
Phone: 915854385
email: plopez@iib.uam.es

SUPLEMENTARY METHODS

Animals and Experimental Design

All animal handling protocols were performed by specialized personnel and approved by the ethical committee of the Institute of Biomedical Research “Alberto Sols” and the Community of Madrid, complying with national (R.D. 53/2013) and European Community guidelines (2010/62/UE).

Eight-week old, healthy male mice of the C57BL6/J strain (n=60, 22±2g) were purchased (Charles River, France), and acclimated for a week to the environmental conditions of the animal quarters of our Institute (Reg. No. ES280790000188). Mice were randomly distributed among the four experimental groups. Animals were identified individually with ear punches and kept in independent cages containing 3-4 mice/cage, in a temperature (21-23°C) and humidity (47%) controlled room, following 12 hours day/night cycles starting at 8:00a.m. Individual body weight measurements were performed every three days. MRI and phenotyping studies were performed with fed *ad libitum,* and 16h fasted, animals. The investigator was not blinded to the group allocation during the experiment. Mice submitted to 16h-fasting were employed for blood

collection and euthanized after the MRI session, while an independent cohort of fed animals was used for the same invasive measurements.

In the phenotyping studies, after the 16h-fasting period, blood was extracted from the venous sinus orbital and used for determinations of the endocrine profile. Two separate animal cohorts (n=10 HFD, n=10 SD) were used to gather the invasive measurements in the fed state, including HRMAS of hypothalamic, hippocampal and cortical biopsies, and blood hormonal profiles (Supplementary Fig. 1).

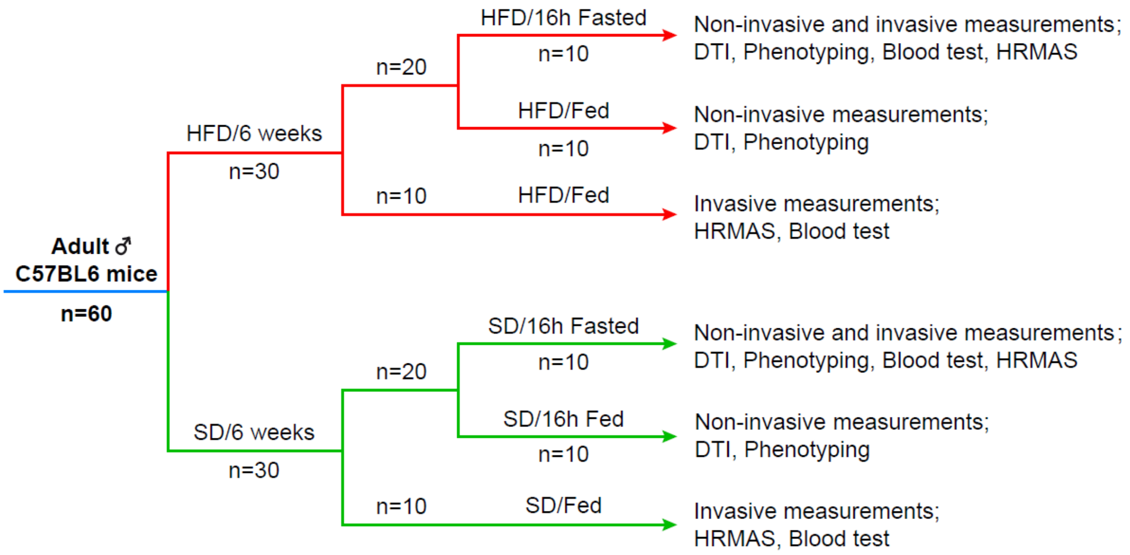

Supplementary Figure 1. Experimental design. Summary of the experimental design indicating the animals involved (n) in HFD, or SD feeding, under fed or fasted conditions. The figure shows the workflow and techniques used in the characterization of the different experimental groups.

Physiological Characterization

Phenotyping. Mice (n=10 HFD, n=10 SD) were individually isolated in high-performance metabolic cages, enabled to monitor, automatically, parameters including: food intake and water consumption, locomotor activities (distance walked-DistK), and respiratory exchange rate (RER). Food intake and water consumption were determined with high precision weight and volume sensors adapted to the cages. DistK was measured using infrared light beams recognizing locomotor activity. RER represents the ratio between CO_2 produced and O_2 consumed, as measured using infrared and paramagnetic gas analyzers, respectively. RER values provide

information on the dominant metabolic substrate consumed with RER 0.7 revealing dominant fat consumption, RER 1.0 indicating dominant carbohydrate consumption, and intermediate RER values reflecting the consumption of mixed diets. To ensure a proper acclimation to the modified environment during phenotyping sessions, all mice were placed in the phenotyping room 48h prior to the recordings. Subsequently, parameters were collected every 1h, for the next 90h, including 74h of free access to food and water, followed by a 16h-fasting period with unrestricted water access.

Endocrine Profile. Blood samples were collected from the venous sinus orbital of anesthetized mice using capillaries and transferred to polypropylene tubes. Plasma was isolated and stored at -80°C until analysis. Citrate was used as anticoagulant throughout. Protease inhibitors (Sigma-Aldrich's DPPIV Inhibitor / Protease Inhibitor cocktails) were added to each tube and centrifuged for 5 minutes at 1200rpm. Appetite related hormones were determined with the MILLIPLEX MAP Mouse Metabolic Hormone Magnetic Bead Panel (MMHMAG-44K Multiplex-assay, Merck, Madrid, Spain), using the Luminex® platform (LabClinics, Madrid, Spain), calibrated with hormone standards of known concentration. Leptin, insulin, ghrelin, PYY, glucagon and GLP-1 concentrations were determined in plasma samples obtained from HFD or SD mice, under the fed or fasted conditions.

Magnetic Resonance Imaging and Spectroscopy

***Diffusion Tensor Imaging**. Equipment.* MRI was performed on a preclinical Bruker 7T BioSpec magnet (16cm bore) equipped with a 90mm gradient insert (360mT/m) and a 23mm volume resonator, interfaced with an Avance III radiofrequency console running under ParaVision 5.1 software (Bruker Biospin, Ettlingen, DE). Procedures started every day at 8:00a.m. imaging two animals per morning to minimize uncertainties derived from varying circadian rhythms in longer acquisitions. This protocol was reproduced until all animals were examined. Before MRI, all animals underwent body weight measurements and blood glucose levels were. Anesthesia was then induced by delivering (1mL/min) an (2-2.5%) isoflurane/oxygen mixture. Mice were subsequently immobilized in a home-made polymethylmethacrylate holder, with a bite-bar coupled to a nose cone mask, and an (1-1.5%) isoflurane/oxygen mixture delivered (1mL/min) as maintenance anesthesia throughout the imaging period. During acquisitions, body temperature of

the animals was kept approximately at 37°C with a thermostatic water blanket, with the level of anesthesia adjusted through the imaging period to maintain respiration rate between 40-60 breaths/ minute. The physiological status of the animal was monitored continuously with a small animal ECG/Temperature device (Model 1025, SA Instruments, Inc. NY) providing respiration rate measurements through a pneumatic pillow placed underneath the animal abdomen.

MRI sequences. MRI protocols included T_2 morphological RARE acquisitions, with the following parameters: TR=2500ms, TE=44ms, 6 averages, RARE factor=8, slice thickness=1mm and in-plane resolution=0.086x0.086mm^2/pixel in the axial orientation. The section containing hypothalamus was identified easily using anatomical coordinates as the slice containing the hypophysis, the third ventricle (3v) and the dorsal third ventricles (D3v) (1), corresponding to the slice located -1.46 mm from Bregma (2,3). Diffusion weighted images used a DTI sequence with diffusion gradients applied in 6 directions, and the following parameters: Δ/δ=20/4ms, TR=2500ms, TE=43ms, slice thickness=1.5mm, in-plane resolution 0.172x0.172mm^2/pixel in axial orientation and two b values of 200 and 1000s/mm^2.

Image analysis. DTI were processed and analyzed with a homemade software package implemented in MatLab (R2010b, the MathWorks Inc., Natick, MA) by fitting in a voxel by voxel based, the diffusion signal decay to a mono-exponential model according to Eq. 1.

$$S(b) = S_0\, e^{-b.\boldsymbol{D}} \qquad \text{[Eq. 1]}$$

where *S(b)* represents the individual voxel intensity in the presence of the diffusion gradient, and S_0 is the individual voxel intensity in the absence of diffusion gradients (b=0s/mm^2). ***D*** is a symmetric tensor named *diffusion tensor*, describing the diffusion of water molecules in the three orthogonal dimensions, with its eigenvalues allowing for the calculation of MD and FA values as indicated by Eqs. 2-3;

$$MD = \frac{\lambda_1+\lambda_2+\lambda_3}{3} \qquad \text{[Eq. 2]}$$

$$FA = \frac{\sqrt{3[(\lambda_1-\langle\lambda\rangle)^2+(\lambda_2+\langle\lambda\rangle)^2+(\lambda_3+\langle\lambda\rangle)^2]}}{\sqrt{2(\lambda_1^2+\lambda_2^2+\lambda_3^2)}} \qquad \text{[Eq. 3]}$$

where λ_1, λ_2 and λ_3 are the eigenvalues of the diffusion tensor. A main restriction of r^2>0.65 was used in the fittings.

High Resolution Magic Angle Spinning ^1H Spectroscopy (^1H HRMAS). Brain samples prepared by microwave fixation were dissected to isolate biopsies from hypothalamus, hippocampus, and Ctx. Briefly, small pieces (10-15mg) of every biopsy were placed in a HRMAS zirconium rotor (4mm OD) fitted with a 50µl cylindrical insert, filled with D_2O and transferred to the HRMAS probe. ^1H HRMAS spectra were acquired at 4°C and 5kHz spinning rate, using an 11.7T Bruker magnet interfaced with an AVANCE radiofrequency console operating at 500.13MHz for ^1H (Bruker Biospin, Wissembourg, FR). Carr-Purcell-Meiboom-Gill (CPMG) spectra were acquired using a spin-echo sequence with a total echo time of 36ms, involving 32K data points in the time domain and 128 scans, suppressing the dominant water resonance with a presaturation pulse (5s).

The resulting spectra were quantified using the LCModel. The program yields values for relative metabolite concentrations and the estimated percentage of standard deviation in the fitting of every metabolite. The model set of metabolite spectra included; acetate (Ac), alanine (Al), aspartate (Asp), choline (Cho), creatine (Cr), phosphocreatine (PCr), γ-aminobutyrate (GABA), glucose (Glc), glutamine (Gln), glutamate (Glu), reduced glutathione (GSH), glycine (Gly), glycerophosphocholine (GPC), guanidoacetate (gAc), isoleucine (Leu), *myo*-inositol (Myo), lactate (Lac), leucine (Leu), N-acetylaspartate (NAA), phosphoethanolamine (PE), phosphocholine (PC), taurine (Tau), threonine (Thr), and valine (Val) (4). Additionally, resonances from the methyl (0.9 ppm) and methylene (1.3 ppm) groups of sufficiently mobile fatty acid droplets (5), were fitted (Lip13 and Lip09, respectively). Quantification of metabolites was assessed by determining their relative concentration to the total creatine (Cr+PCr) content in the sample. Only metabolites with Cramér–Rao lower bound values smaller than 20% were considered.

Statistics

Univariate Analysis. Physiologic and phenotyping statistics were calculated using GraphPad Prism 6 (GraphPad Software, San Diego, CA). Body weight differences between diet groups during the 6-week period of controlled feeding, were evaluated with multiple unpaired t-tests and corrected for multiple comparisons using the Holm-Sidak test. Body weight and blood glucose values before the MRI evaluations (fed or fasted) were compared either with two-tailed paired t-

tests (for the fed vs. fasted evaluations), or with multiple unpaired t-tests (SD vs HFD comparisons) with the Holm-Sidak method. Blood hormones, MRI and HRMAS statistical analyses, were performed using the IBM SPSS package (IBM SPSS Statistics for Windows, Version 25.0. Armonk, NY, IBM Corp). Differences in blood hormone concentrations were investigated using the UNIANOVA method.

Generalized Linear Model. MD and FA statistical differences were analyzed using the generalized linear model (GzLM) with generalized estimating equations (GEE), to compare values of all animals in the different experimental conditions. Briefly, an *ad hoc* GEE model linearly relates the dependent variables (calculated MD and FA) to the independent parameters (diet and feeding condition), allowing for the analysis of repeated measures (regions and voxels). Subsequently, the effect of the independent parameters on the calculated estimates was evaluated statically using the Wald test. To evaluate statically HRMAS and blood hormones data, a logarithmic transformation of the values was calculated prior to the statistical evaluation for homogenizing purposes (6). A linear mixed model (LMM) was used to evaluate the HRMAS transformed data. In all cases, p values<0.05 were considered statistically significant.

Z-Score Evaluation. Z-Scores from MRI parameters, brain metabolites measured in HR-MAS, blood determinations and phenotypic measurements were calculated in the SD-fed, SD-fasted, HFD-fed, HFD-fasted conditions, using SPSS software as described in Eq. 4.

$$Zscore_i = \frac{x_i - \bar{x}}{s} \qquad \text{[Eq. 4]}$$

where $x_i$represents the mean value of a variable in a specific diet and feeding condition, $\bar{x}$ accounts for the sample mean between the four conditions, and S the sample standard deviation. Z-Scores measure the distance from the mean value. Under these conditions, a Z-Score value of 0 indicates no deviation from the mean, while Z-Scores of e.g. ±1 or ±2, reveal that the mean value of the variable, lies 1 or 2 standard deviations above or below, the mean. Z-Scores provide therefore, a robust procedure to standardize all measurements of a data set in a uniform scale.

Z-Scores from all variables were then represented in four independent bar graphs corresponding to SD-fed, SD-fasted, HFD-fed and HFD-fasted conditions, allowing for an overview of the patterns of change of groups of variables reacting similarly to the different experimental conditions (Figure 5). Variables that presented more than two Z-scores below 0.5

where not considered relevant, and were not further included in the bar graphs, to facilitate the qualitative comparisons.

ALSCAL. It is a multidimensional scaling, which attempts to find the structure in a set of distance measures between objects or cases. This task is accomplished by assigning observations to specific locations in a conceptual space (usually two- or three-dimensional) such that the distances between points in the space match the given dissimilarities as closely as possible. The distances are calculated with the Euclidean distance expression:

$$d(P,Q) = \sqrt{\sum_{i=0}^{n}(p_i - q_i)^2} \qquad \text{[Eq. 5]}$$

where P and Q are the objects, p_i and q_i are their respective coordinates. The Euclidean distance allows measuring the distance between two objects in a n-dimensional space. Then, the distances are transforming to coordinates in a new two or three dimensional space. The differences between the values in the present parameters, from values in the order of thousands to values with only decimals, caused that this method was applied in the Z-Score values previously calculated, which allows comparing the values in a proper way.

In the present study, first it was calculated the Euclidean distance between the Z-Score of the original variables (MD, FA, GABA, Glu, Myo, NAA, Glc, GPC+Cho, Lip09, Lip13, relation Lip1.3/0.9, glucagon, insulin, leptin, PYY, weight, glucose, drink, food, RER, DistK and calories), as measured in the four experimental conditions (SD-fed, SD-fasted, HFD-fed, HFD-fasted), The calculus was made using the SPSS software algorithm Proximities. Then, the transformation into a new representation space with only two dimensions was made with ALSCAL algorithm in SPSS, with a Euclidean model.

In all cases, the variance was similar among the groups that were statistically compared.

SUPPLEMENTARY RESULTS

Physiological and Imaging Characterization of the Animal Model

Table 1. Mean and standard deviation values of body weight (g) and blood glucose levels (mg/dL) of mice cohorts for each state and feeding condition.

**Diet/Condition**	**Body weight (g)**	**Blood glucose levels (mg/dL)**

SD-fed	30±1.1	152.6±19.2
SD-fasted	26.9±1.1	108.1±22.7
HFD-fed	32.7±2	185.9±42.2
HFD-fasted	30.7±2.1	127.9±33.7

Table 2. Mean and standard deviation values of MD and FA of mice cohorts for each region, state and feeding condition.

Diffusion parameter	Region	Diet	Condition	Mean	Standard Deviation
MD ($\mu m^2/s$)	Cortex	SD	Fed	641. 5	64.7
			Fasted	666.0	100.1
		HFD	Fed	750.7	87.6
			Fasted	698.4	122. 5
	Hippocampus	SD	Fed	665.3	79.1
			Fasted	671.7	94.3
		HFD	Fed	817.1	150.7
			Fasted	750.0	159.4
	Hypothalamus	SD	Fed	654.3	108.3
			Fasted	653.6	111.7
		HFD	Fed	726.5	141.4
			Fasted	751.0	155.7
	Brain	SD	Fed	740.2	162.3
			Fasted	705.1	118.9
		HFD	Fed	815.3	148.8
			Fasted	795.1	183.5
FA	Cortex	SD	Fed	0.31	0,17
			Fasted	0.31	0,16
		HFD	Fed	0.37	0,11
			Fasted	0.41	0,12
	Hippocampus	SD	Fed	0.28	0,14
			Fasted	0.30	0.14
		HFD	Fed	0.37	0.14
			Fasted	0.44	0.13
	Hypothalamus	SD	Fed	0.38	0.17
			Fasted	0.33	0.13
		HFD	Fed	0.31	0.14
			Fasted	0.36	0.14
	Brain	SD	Fed	0.31	0.16
			Fasted	0.31	0.15
		HFD	Fed	0.39	0.14
			Fasted	0.42	0.15

^{1}H-HRMAS Spectroscopy

Representative HRMAS spectra from the different diets/feeding conditions are shown in Suppl. Fig. 2

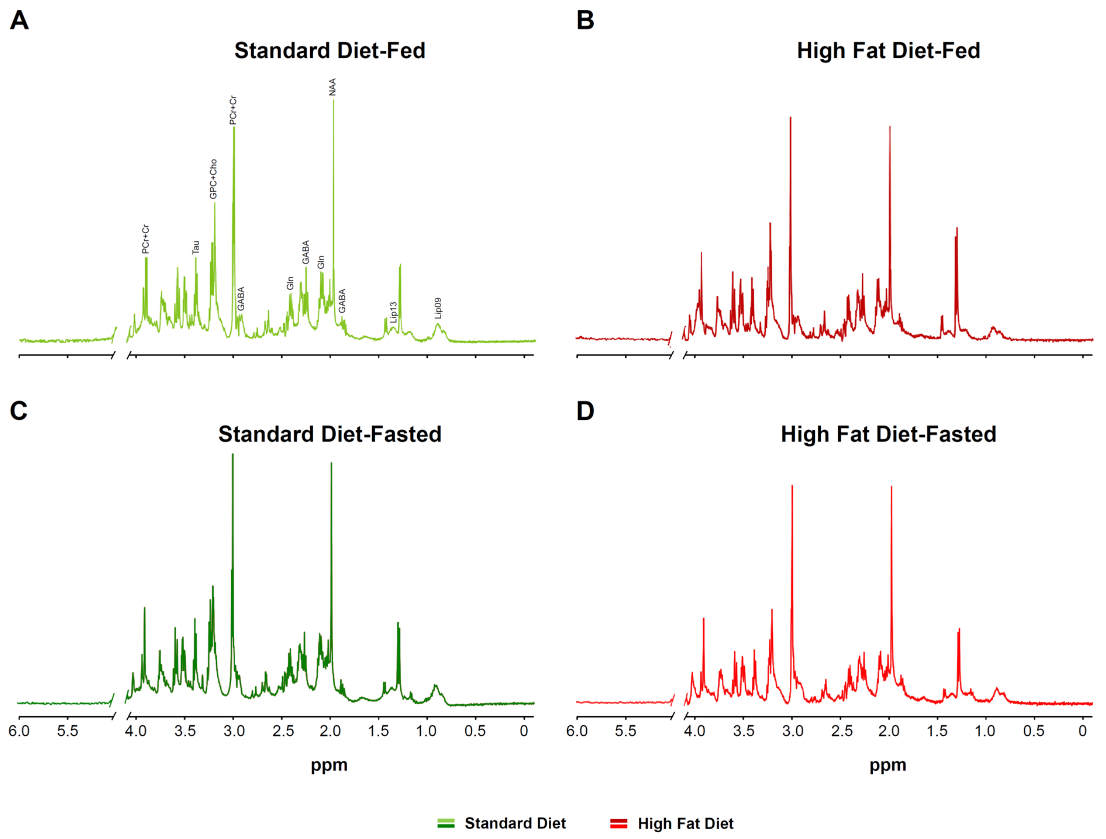

Supplementary Figure 2. Representative HRMAS spectra of hypothalamic biopsies from mice subjected to HFD or SD diets, under fed/ fasted conditions. Lip 09: methyl resonance from saturated fatty acids (0.9 ppm), Lip13: methylene resonance from saturated fatty acids (1.3 ppm), NAA: N-acetyl-aspartic acid, Glu: glutamate, Gln: glutamine, GABA: γ-aminobutyric acid, Glc: glucose, Tau: taurine, Myo: *myo*-inositol. Note that the region 5-6 ppm shows no vinyl resonances indicating that the fatty acid resonances at 0.9 and 1.3 ppm are derived from saturated fatty acids.

Regional ASCAL Analysis

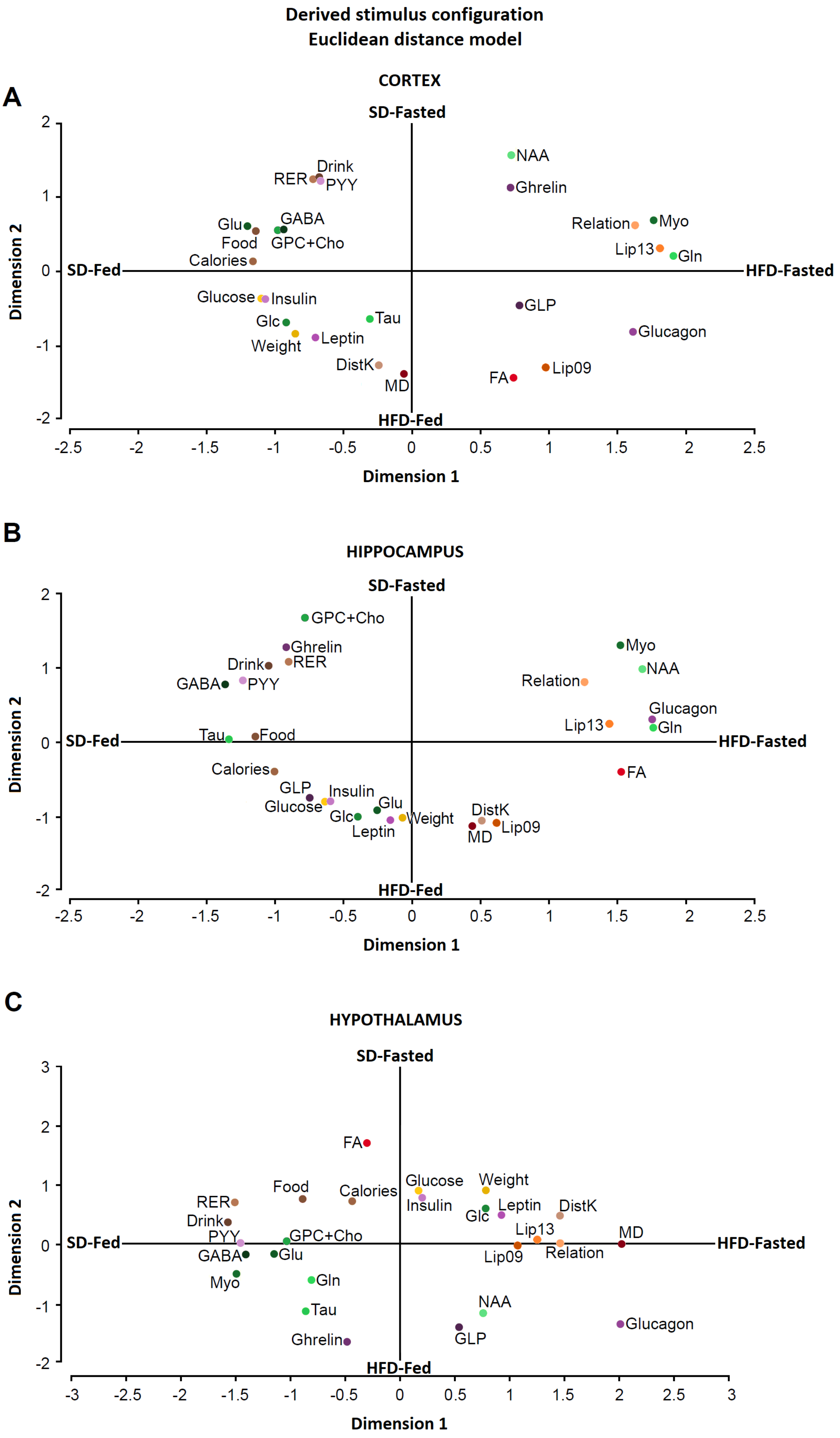

Derived stimulus configuration
Euclidean distance model
A
CORTEX
SD-Fasted
HFD-Fed
SD-Fed
HFD-Fasted
Dimension 1
Dimension 2
NAA
Ghrelin
Drink
RER
PYY
Glu
GABA
Food
GPC+Cho
Calories
Relation
Myo
Lip13
Gln
Glucose
Insulin
Glc
Tau
Weight
Leptin
GLP
Glucagon
DistK
MD
FA
Lip09
B
HIPPOCAMPUS
SD-Fasted
HFD-Fed
SD-Fed
HFD-Fasted
Dimension 1
Dimension 2
GPC+Cho
Ghrelin
Drink
RER
GABA
PYY
Myo
NAA
Relation
Glucagon
Lip13
Gln
Tau
Food
FA
Calories
GLP
Insulin
Glucose
Glu
Glc
Weight
Leptin
DistK
Lip09
MD
C
HYPOTHALAMUS
SD-Fasted
HFD-Fed
SD-Fed
HFD-Fasted
Dimension 1
Dimension 2
FA
Food
Calories
Glucose
Weight
RER
Insulin
Leptin
DistK
Drink
Glc
Lip13
MD
PYY
GPC+Cho
GABA
Glu
Lip09
Relation
Myo
Gln
Tau
NAA
Glucagon
Ghrelin
GLP

Supplementary Figure 3. ALSCAL diagram representations from different cerebral regions. A: cortex, B: hippocampus and C: hypothalamus. Variables are displayed within the new coordinate space, with each axis representing a different diet-feeding condition. Each variable is represented with respect to the rest of the variables by the transformation of the Euclidean distance between each pair of variables. Abbreviation and colors are equivalent to those showed in Fig. 6.

SUPPLEMENTARY BIBLIOGRAPHY

1. Suzuki K, Simpson KA, Minnion JS, Shillito JC, Bloom SR. The role of gut hormones and the hypothalamus in appetite regulation. Endocrine Journal. 2010;57(5):359–72.

2. Lizarbe B, Benítez A, Sánchez-Montañés M, Lago-Fernandez L, Garcia-Martin M, López-Larrubia P, et al. Imaging hypothalamic activity using diffusion weighted magnetic resonance imaging in the mouse and human brain. NeuroImage. 2012 Sep 19;64:448–57.

3. Benítez A, Lizarbe B, Guadilla I, López-Larrubia P, Lago-Fernández LF, Cerdán S, et al. Cerebral hunger maps in rodents and humans by diffusion weighted MRI. Appetite. 2019 Nov 1;142:104333.

4. Duarte JMN, Lei H, Mlynárik V, Gruetter R. The neurochemical profile quantified by in vivo 1H NMR spectroscopy. NeuroImage. 2012 Jun 1;61(2):342–62.

5. Delikatny EJ, Chawla S, Leung D-J, Poptani H. MR-visible lipids and the tumor microenvironment. NMR Biomed. 2011/04/27 ed. 2011 Jul;24(6):592–611.

6. Feng C, Wang H, Lu N, Tu XM. Log transformation: application and interpretation in biomedical research. Statistics in Medicine. 2013 Jan 30;32(2):230–9.
